# Long-term donepezil use for dementia with Lewy bodies: results from an open-label extension of Phase III trial

**DOI:** 10.1186/s13195-014-0081-2

**Published:** 2015-02-03

**Authors:** Etsuro Mori, Manabu Ikeda, Reiko Nagai, Kazutaka Matsuo, Masaki Nakagawa, Kenji Kosaka

**Affiliations:** Department of Behavioral Neurology and Cognitive Neuroscience, Tohoku University Graduate School of Medicine, 2-1, Seiryo-machi, Aoba-ku, Sendai, Miyagi 980-8575 Japan; Department of Neuropsychiatry, Faculty of Life Sciences, Kumamoto University, 1-1-1 Honjo, Chuo-ku, Kumamoto 860-8556 Japan; Eisai Product Creation Systems, Eisai Co., Ltd., 4-6-10 Koishikawa, Bunkyo-ku, Tokyo 112-8088 Japan; Department of Psychiatry, Yokohama City University School of Medicine, 3-9 Fukuura, Kanazawa-ku, Yokohama, Kanagawa 236-0004 Japan

## Abstract

**Introduction:**

The long-term efficacy and safety of donepezil 10 mg in patients with dementia with Lewy bodies (DLB) were investigated in a 52-week Phase 3 trial.

**Methods:**

This 52-week study consisted of 16-week randomized placebo-controlled (RCT) and 36-week open-label extension phases. Of 142 DLB patients enrolled in the RCT phase (three arms: placebo, 5 mg, and 10 mg), 110 entered the extension phase. The placebo group of the RCT phase initiated active treatment at week 16, and the active groups maintained allocated treatment and dosages until week 24. After week 24, all patients received 10 mg. Dose reduction to 5 mg for safety concerns was allowed. Efficacy measures included Mini-Mental State Examination (MMSE) for cognitive function and Neuropsychiatric Inventory (NPI) for behavioral symptoms. Safety evaluations included adverse events (AEs) and the unified Parkinson disease rating scale.

**Results:**

In total, 100 subjects completed the study. Cognitive function improvement was sustained for 52 weeks (MMSE at week 52 in 10 mg: 2.8 ± 3.5 (mean ± standard deviation); *P* <0.001, Student paired *t* test)). Those who received placebo in the RCT phase showed an improvement after starting active treatment. NPI improved in all the groups throughout the study, including the placebo period. In the subgroup of the 5 mg group without remarkable cognitive or behavioral improvement at week 24, further improvement was observed after a dose increase to 10 mg. After week 24, 21 patients experienced dose reduction. The incidence of any AEs did not increase over time.

**Conclusions:**

The long-term administration of donepezil at 10 mg/day improved cognitive function for up to 52 weeks in patients with DLB without increasing the risk of clinically significant safety events.

**Trial registration:**

NCT01278407. Trial registration date: January 14, 2011.

**Electronic supplementary material:**

The online version of this article (doi:10.1186/s13195-014-0081-2) contains supplementary material, which is available to authorized users.

## Introduction

Dementia with Lewy bodies (DLB) is a common form of dementia in the elderly, and constitutes the second largest group of patients with dementia, following Alzheimer disease (AD) [1]. The core clinical features of DLB include neuropsychiatric symptoms and parkinsonism, as well as cognitive impairment characterized by deficits of attention, executive function, and visual perception [2]. The progression of cognitive impairment is faster than or similar to that in AD [3-6]. Patients with DLB have a higher risk for falls [7,8], higher risk of admission [9], lower activities of daily living, lower quality of life, and a heavier caregiver burden [10-13], compared with those with AD.

Cholinergic neurotransmission is more defective in patients with DLB than in those with AD [14]. Although cholinergic losses in DLB affect both brainstem and basal forebrain presynaptic nuclei, postsynaptic cortical muscarinic and nicotinic receptors are preserved [15]. For these reasons, cholinesterase inhibitors (ChEIs) may be effective for treating DLB, and several clinical trials have demonstrated favorable potential of ChEls such as galantamine, rivastigmine, and donepezil for DLB [16-22].

The previous Phase 2, 12-week, randomized double-blind placebo-controlled trial of three different doses of donepezil in patients with DLB [22] demonstrated that donepezil significantly improved all of the efficacy end-points of cognitive impairment, behavioral and psychiatric symptoms, global clinical symptoms, and caregiver burden, compared with placebo, and the open-label 1-year extension study of donepezil at 5 mg/day [23] showed that the major concerns about the safety of long-term administration of 5 mg donepezil, including parkinsonism and cardiovascular events, were minimal, and that the mild improvement of cognitive impairment and psychiatric symptoms was sustained for up to 52 weeks.

Based on these results, a Phase 3 study, which integrated a randomized placebo-controlled, double-blind comparative study (RCT phase) and an open-label extension study (extension phase), was conducted in patients with DLB to confirm the superiority of donepezil at 5 and 10 mg/day for 12 weeks over placebo and to evaluate the safety and efficacy of long-term administration of 10 mg/day. The RCT phase yielded the efficacy of donepezil on cognitive impairment with significant improvement in MMSE compared with placebo in the 10 mg group (mean ± standard deviation (SD): 0.6 ± 3.0 and 2.2 ± 2.9 in the placebo and 10 mg group, respectively; *P* = 0.016, analysis of covariance (ANCOVA)), although a significant difference was not detected on the behavioral and neuropsychiatric measures (change in Neuropsychiatric Inventory-2 (NPI-2) (mean ± SD): −2.0 ± 4.2 and −2.9 ± 4.7 in the placebo and 10 mg group, respectively; *P* = 0.391, ANCOVA), falling short of confirming the pre-defined superiority of donepezil compared with placebo at either dose (5 or 10 mg/day). With detailed information of the results reported elsewhere [24], this report describes the results obtained through long-term administration of the higher dose of donepezil in DLB.

## Methods

### Patients

Patients diagnosed as probable DLB, according to the consensus diagnostic criteria [2], were recruited from 72 psychiatric or neurologic specialty centers throughout Japan from February 2011 to March 2012. Eligible patients were outpatients aged ≥50 years with mild to moderately severe dementia (10 to 26 on the MMSE and Clinical Dementia Rating ≥0.5) and behavioral and psychiatric symptoms NPI-plus ≥8 and NPI-2 ≥ 1). NPI-plus consisted of 12 items: original 10 items [25,26], sleep, and cognitive fluctuation, which was reported as Cognitive Fluctuation Inventory [27]. NPI-2 consisted of hallucinations and cognitive fluctuation [22]. Caregivers of the eligible patients had to stay with them routinely at least 3 days per week and 4 hours per day, provide information for this study, assist with the compliance with treatment, and escort them to required visits. The evidence or rationale for the presence of the core features, on which each diagnosis of DLB was based, was provided and examined by the review board (Mori, Ikeda, and Kosaka) to assure the validity of the diagnosis.

Exclusion criteria included Parkinson disease diagnosed at least 1 year prior to the onset of dementia; focal vascular lesions on MRI or CT that might cause cognitive impairment (for example, infarcts/hemorrhages affecting the thalamus, caudate nucleus, or globus pallidus, single infarct of diameter ≥1.5 cm or multiple infarcts in any other regions, and moderate or severe white matter changes); other neurologic or psychiatric diseases; clinically significant systemic disease; complications or history of severe gastrointestinal ulcer, severe asthma or obstructive pulmonary disease; systolic hypotension (<90 mm Hg); bradycardia (<50 m^−1^); sick sinus syndrome; atrial or atrioventricular conduction block; QT-interval prolongation (≥450 ms); hypersensitivity to donepezil or piperidine derivatives; severe parkinsonism (Hoehn and Yahr score ≥ IV) [28]; and treatment with ChEIs or any investigational drug within 3 months before screening. ChEIs, antipsychotics, and anti-Parkinson drugs other than L-dopa or dopamine agonists were not allowed during the study.

### Procedures

**Figure 1:**
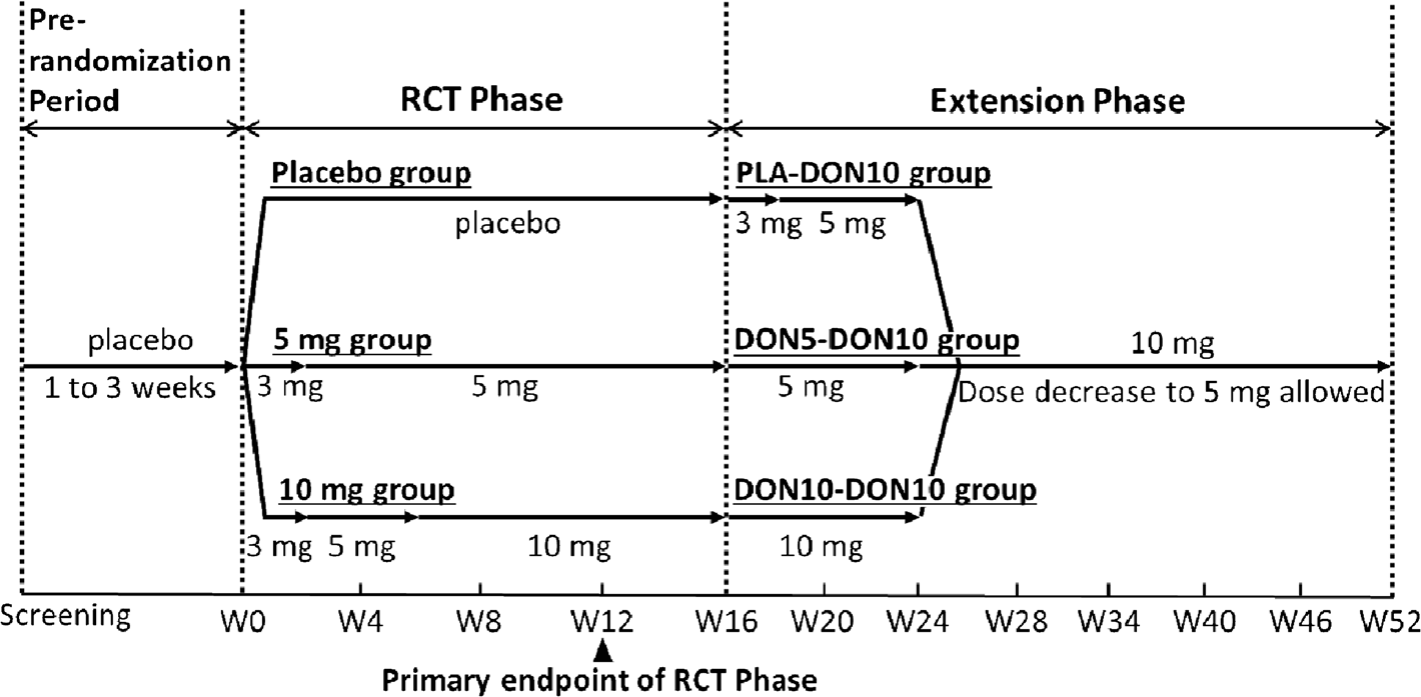
**Study flow.** RCT, randomized placebo-controlled.

This was a 52-week, multicenter, Phase 3 study consisting of a 16-week, randomized, double-blind, placebo-controlled phase (referred as RCT phase) and the subsequent 36-week, open-label extension phase (referred as extension phase) (Figure 1).

After a 2-week prerandomization period with placebo administration, the patients were randomly assigned in a 1:1:1 ratio to placebo or 5 mg or 10 mg of donepezil in the RTC phase. Treatment began with 3 mg and was then titrated. After the RCT phase (ended before Week 16), the dose was maintained until Week 52 in the 10 mg group of the RCT phase (referred to as DON10-DON10). In the 5 mg group of the RCT phase, the dose was increased to 10 mg/day at Week 24 (referred to as DON5-DON10). The placebo group started active treatment with 3 mg at the beginning of the extension phase (at Week 16), and the dose was then increased to 5 mg at Week 18 and to 10 mg at Week 24 (referred to as PLA-DON10). After Week 24, dose reduction to 5 mg was allowed if continuation at 10 mg caused any safety concerns.

The randomization code was broken in August 2012 after all data of the RCT phase were fixed before the end of the extension phase (March 2013). The physicians and patients were kept blinded to the treatment allocation until the extension phase completion by blinded titration by using a similar placebo.

Written informed consent was obtained from the patient (if at all possible) and his/her primary caregiving family member before initiating the study procedures. The study was conducted in accordance with the principles of the Declaration of Helsinki. The protocol was approved by the institutional review board at each center (see Additional file 1).

### Outcome measures

Cognitive function was assessed by using the MMSE [29]. Behavioral and psychiatric symptoms were assessed by using the NPI-2 [22] and NPI-10 [25,27]. NPI-2 was calculated as the sum of the scores for hallucinations and cognitive fluctuation [26], which correspond to the core features of DLB in the consensus criteria. These measures were assessed at Weeks 0, 4, 8, 12, 16, 20, 24, 28, 34, 40, 46, and 52. Caregiver burden was assessed by using the Zarit Caregiver Burden Interview (ZBI) [30], which evaluates the physical, psychological, and social consequences of caring activities. The ZBI contains 22 items scored from 0 (best) to 4 (worst), from which a total score of 0 to 88 is calculated. The ZBI was assessed at 0, 12, 24, 40, and 52 weeks.

Safety was assessed based on the adverse events (AEs), vital signs, electrocardiogram, and laboratory tests. All AEs were classified and coded according to Medical Dictionary for Regulatory Activities (MedDRA) terms. Gastrointestinal symptoms, parkinsonian symptoms, psychiatric symptoms, and arrhythmia were assessed as AEs of interest. Motor function was assessed as a safety measure by using the Unified Parkinson’s Disease Rating Scale (UPDRS) part III [31].

### Statistical analyses

Sample-size calculation is reported elsewhere [24]. The safety analysis set (SAS) comprised all patients who received at least one dose of donepezil and had safety-assessment data. The incidence of AEs was summarized based on the treatment period with the active drug; safety analysis in the DON5-DON10, DON10-DON10 groups, and the combined group of them (referred to as DON-DON10) encompasses the entire study period, including the RCT phase (52 weeks), and that in the PLA-DON10 group covers the extension phase alone (36 weeks). Laboratory parameters and vital signs were summarized by descriptive statistics. Scores or their changes in UPDRS part III from the baseline in each of the DON5-DON10 and DON10-DON10 groups or in the DON-DON10 group were analyzed by using Student paired *t* test.

Efficacy was analyzed in the full analysis set (FAS), including the randomized patients who received the study drug at least once and had valid efficacy assessment data at more than one point. Exploratory analyses were performed as appropriate to compare scores at every evaluation point in each of the three groups with the baseline (Week 0) by paired *t* tests, and in the DON5-DON10 group, also to compare scores at every evaluation point with Week 24 to evaluate the effect of dose increment by paired *t* tests and mixed-effect model for repeated measures (MMRMs). The parameters included in the model were the Observed value at week 24 as a covariate, and Subgroup stratified according to the degree of improvement, Visit, and Interaction as factors. Values at the final evaluation were imputed by using a last observation carried forward (LOCF) method.

*P* values were not adjusted for multiplicity. All statistical tests were two-tailed, and *P* < 0.05 was considered to indicate statistical significance. All analyses were made on SAS versions 9.1 and 9.2 (SAS Institute, Cary, NC, USA).

## Results

### Baseline characteristics

**Figure 2:**
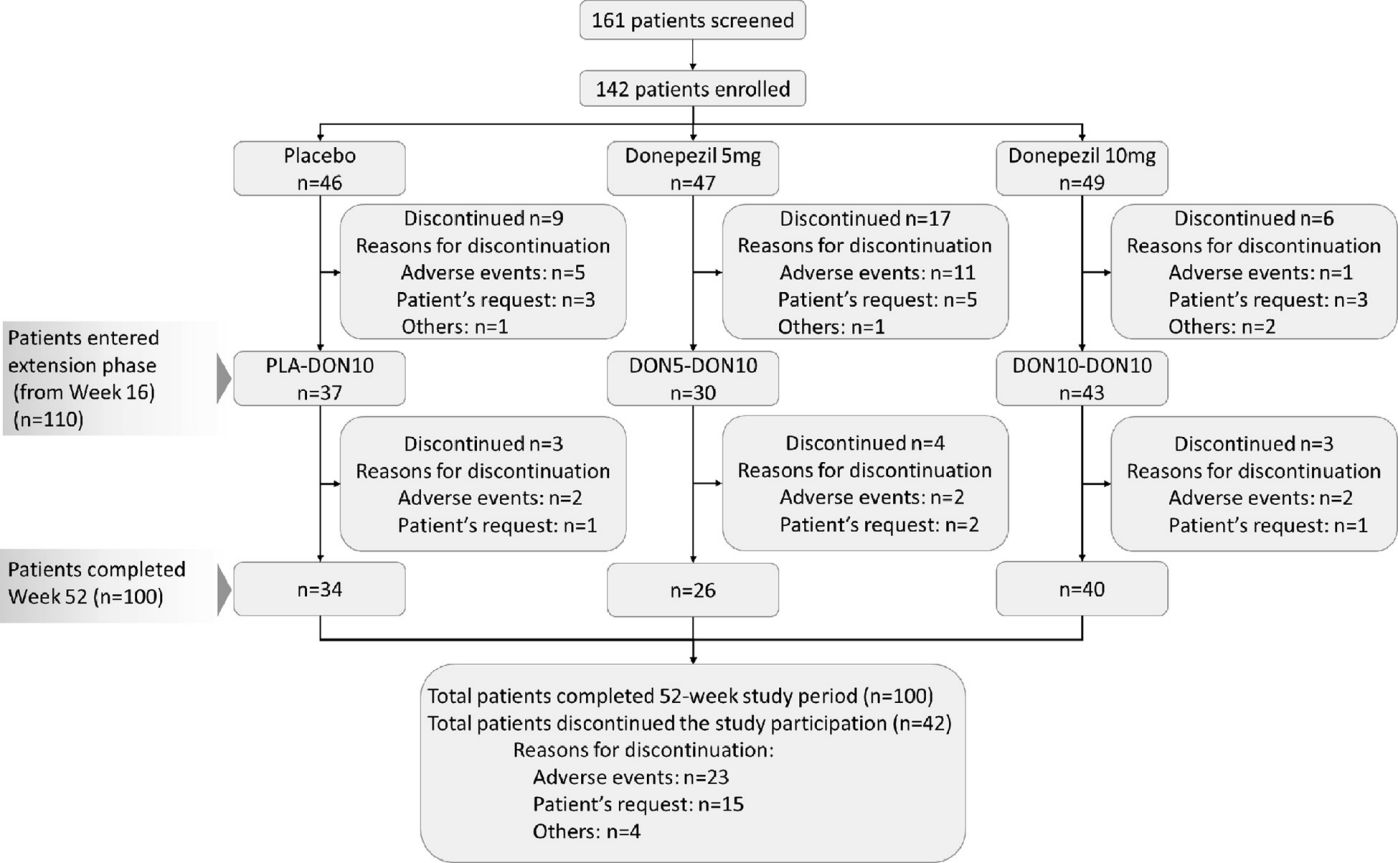
**Patient disposition.**

Of 161 patients enrolled in the pre-randomization period, 142 were enrolled in the RCT phase and randomized to the placebo, 5 mg, and 10 mg groups (46, 47, and 49 patients, respectively). During the RCT phase (by Week 16), 32 patients were discontinued (9, 17, and 6 patients in the placebo, 5 mg, and 10 mg groups, respectively). The reasons for the discontinuations were AEs (17 patients), patient’s request (11 patients), and other reasons (4 patients). In the placebo group, 37 patients started active treatment at Week 16. During the extension phase, 10 patients were discontinued (3, 4, and 3 patients in the PLA-DON10, DON5-DON10, and DON10-DON10 groups, respectively) because of AEs (6 patients) and patient's request (4 patients) (Figure 2).

Demographic and baseline characteristics of the FAS are summarized in Table 1. No characteristic differences occurred between the three groups. Females accounted for 58.0%. The mean age was 77.9 (range, 57 to 95) years; all but 2 patients were 65 years or older. Dementia medication had previously been used by only 5.8% of the patients. The mean score of the MMSE at baseline was 20.4 points.

**Table 1:** **Patient demographics and baseline characteristics (FAS)**

	**PLA-DON10**	**DON5-DON10**	**DON10-DON10**	**DON-DON10**
	**(** ***n*** **= 37)**	**(** ***n*** **= 45)**	**(** ***n*** **= 49)**	**(** ***n*** **= 94)**
Sex, number (%)				
Male	14 (37.8)	20 (44.4)	21 (42.9)	41 (43.6)
Female	23 (62.2)	25 (55.6)	28 (57.1)	53 (56.4)
Age, years	76.7 ± 6.0	78.8 ± 5.1	77.7 ± 6.8	78.2 ± 6.1
Weight, kg	51.52 ± 10.68	50.68 ± 9.24	51.72 ± 9.89	51.22 ± 9.55
Duration of dementia, years	2.1 ± 2.4	2.7 ± 1.8	2.3 ± 1.9	2.5 ± 1.9
History of antidementia medication, number (%)				
Yes	1 (2.7)	3 (6.7)	4 (8.2)	7 (7.4)
No	36 (97.3)	42 (93.3)	45 (91.8)	87 (92.6)
Visual hallucinations, number (%)				
Yes	37 (100.0)	39 (86.7)	39 (79.6)	78 (83.0)
No	0	6 (13.3)	10 (20.4)	16 (17.0)
Cognitive fluctuation, number (%)				
Yes	34 (91.9)	41 (91.1)	46 (93.9)	87 (92.6)
No	3 (8.1)	4 (8.9)	3 (6.1)	7 (7.4)
Parkinsonism, number (%)				
Yes	32 (86.5)	39 (86.7)	44 (89.8)	83 (88.3)
No	5 (13.5)	6 (13.3)	5 (10.2)	11 (11.7)
Hoehn & Yahr, number (%)				
I	4 (10.8)	8 (17.8)	7 (14.3)	15 (16.0)
II	15 (40.5)	17 (37.8)	19 (38.8)	36 (38.3)
III	13 (35.1)	14 (31.1)	18 (36.7)	32 (34.0)
MMSE	20.2 ± 4.3	20.6 ± 4.1	20.3 ± 4.8	20.4 ± 4.4
NPI-2	6.9 ± 3.9	6.9 ± 4.5	7.3 ± 4.7	7.1 ± 4.6
NPI-10	19.1 ± 13.5	18.9 ± 15.3	16.6 ± 11.7	17.7 ± 13.5
ZBI	26.0 ± 15.4	28.3 ± 18.5	31.4 ± 17.8	29.9 ± 18.1

FAS, full analysis set, MMSE, Mini-Mental State Examination, NPI: Neuropsychiatric Inventory, ZBI: Zarit Caregiver Burden Interview. Values are expressed as mean ± SD, unless otherwise specified.

### Cognitive function

**Figure 3:**
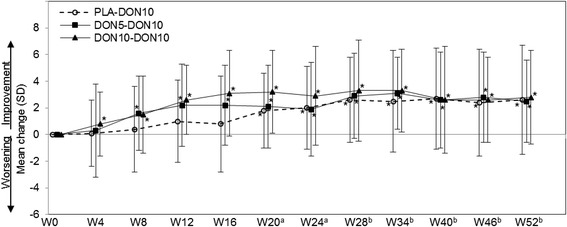
**Mean change in MMSE from baseline (FAS).** MMSE, Mini-Mental State Examination; FAS, full-analysis set. ^**(a)**^ PLA-DON10 group started treatment with 3 mg at Week 16, and the dose was increased to 5 mg at Week 18. ^**(b)**^ PLA-DON10 and DON5-DON10 groups started treatment with 10 mg at Week 24 (dose decrease to 5 mg was allowed). *P < 0.05 (paired *t* test versus Week 0).

Changes in MMSE are shown in Figure 3. Significant improvement compared with baseline was observed from Weeks 8 to 52 in the DON5-DON10 group, and from Week 4 to 52 in the DON10-DON10 group. The mean changes (mean ± SD, Student paired *t* test) at Week 52 and at the final evaluation (LOCF) from baseline were 2.5 ± 3.1 (*P* < 0.001) and 1.3 ± 3.6 (*P* = 0.018) in the DON5-DON10 group, 2.8 ± 3.5 and 2.4 ± 3.7 (*P* < 0.001 each) in the DON10-DON10 group, respectively.

**Figure 4:**
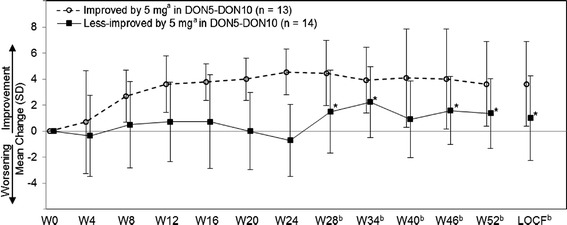
**Mean MMSE change in subgroups of improved and less-improved by 5 mg (FAS, DON5-DON10 group).** MMSE, Mini-Mental State Examination; FAS, full analysis set; LOCF, last observation carried forward. ^**(a)**^The cognitively improved by 5 mg is defined as a patient with 3 points or more improvement in the MMSE score at Week 24, and the less-improved as a patient with fewer than 3 points improvement. ^**(b)**^Treatment with 10 mg started at Week 24 (dose decrease to 5 mg was allowed). *P < 0.05 (paired *t* test versus Week 24).

In the DON5-DON10 group, MMSE increased by 0.4 to 1.1 points at Week 28 to 52 compared with that before the dose increase at Week 24, although it was not significant (Student paired *t* test). For further exploration of this result, changes in MMSE by the subgroups with and without MMSE improvement of 3 points or more from baseline at Week 24 (cognitively improved and less improved by 5 mg) were calculated (Figure 4). Using MMRM for the observed value at or after Week 24, the effect of dose increment was found significant (subgroup, visit, and interaction were *P* = 0.018, *P* = 0.328, and *P* = 0.047, respectively). In the subgroup of less-improved, MMSE significantly increased after dose increment (mean changes from Week 24 with SD (Student paired *t* test) at Weeks 28, 34, 46, and 52: 2.2 ± 3.1 (*P* = 0.019), 2.6 ± 3.2 (*P* = 0.011), 2.0 ± 2.4 (*P* = 0.013), and 1.8 ± 2.2 (*P* = 0.019), respectively).

The PLA-DON10 group showed significant improvement from the baseline (Week 0) through the period after starting active drug at Week 16; the mean changes at Week 28 or later were similar to those in the DON5-DON10 and DON10-DON10 groups, in which treatment with active drugs was started earlier.

In 18 patients whose dose was reduced from 10 mg to 5 mg because of adverse events (9, 4, and 5 patients in the PLA-DON10, DON5-DON10, and DON10-DON10 groups), the change in MMSE from the last administration of the 10 mg was calculated. The changes (mean ± SD) at 6, 12, 18, and 24 weeks after the dose reduction were 0.7 ± 3.0, 0.5 ± 3.5, −0.5 ± 3.6, and −0.7 ± 3.9, respectively; the score was still above the baseline at 24 weeks after the dose reduction (mean change from the baseline, 1.0 ± 3.8).

### Behavioral and neuropsychiatric symptoms

**Figure 5:**
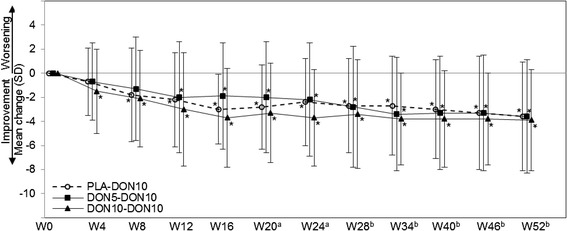
**Mean change in NPI-2 from baseline (FAS).** NPI, Neuropsychiatric Inventory; FAS, full analysis set. ^**(a)**^PLA-DON10 group started treatment with 3 mg at Week 16, and the dose was increased to 5 mg at Week 18. ^**(b)**^PLA-DON10 and DON5-DON10 groups started treatment with 10 mg at Week 24 (dose decrease to 5 mg was allowed). *P < 0.05 (paired *t* test).

NPI-2 significantly improved compared with baseline from Weeks 12 to 52 in the DON5-DON10, and from Weeks 4 to 52 in the DON10-DON10 groups (Figure 5). The mean changes (mean ± SD, Student paired *t* test) at Week 52 and at the final evaluation (LOCF) from baseline were −3.6 ± 4.7 (*P* < 0.001) and −2.1 ± 4.8 (*P* = 0.005) in the DON5-DON10 group, and −3.9 ± 4.2 and −3.4 ± 4.4 (*P* < 0.001 each) in the DON10-DON10 group, respectively. The PLA-DON10 group also showed a sustained reduction in the score from the RCT phase under placebo administration through the extension phase.

**Figure 6:**
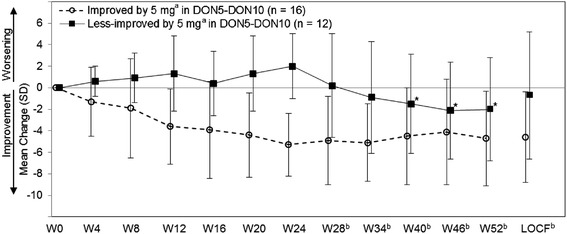
**Mean NPI-2 change in subgroups of improved and less improved by 5 mg (FAS, DON5-DON10 group).** NPI, Neuropsychiatric Inventory; FAS, full analysis set; LOCF, last observation carried forward. ^**(a)**^The behaviorally improved by 5 mg is defined as a patient with 30% or more improvement in NPI-2 score at Week 24, and the less-improved as a patient with less than 30% improvement. ^**(b)**^Treatment with 10 mg started at Week 24 (dose decrease to 5 mg was allowed). *P < 0.05 (paired *t* test versus Week 24).

In the DON5-DON10 group, NPI-2 decreased by 0.6 to 1.0 points at Weeks 28 to 52 compared with that before the dose increase at Week 24, although it was not significant (Student paired *t* test). Changes in NPI-2 by the subgroups with and without NPI-2 improvement of 30% or more from baseline at Week 24 (behaviorally improved and less improved by 5 mg) are shown in Figure 6. As the result of an MMRM for observed value at or after Week 24 with observed value at week 24 as a covariate, and with subgroup, visit and interaction as factors, the factor of interaction were significant (*P* < 0.001) and the factors of subgroup and visit were not significant (*P* = 0.282, *P* = 0.199). In the subgroups of less-improved, NPI-2 significantly decreased after dose increment (mean changes from Week 24 with SD (Student paired *t* test) at Weeks 40, 46, and 52: −3.2 ± 4.0 (*P* =0.033), −3.8 ± 4.9 (*P* = 0.035), and −3.7 ± 4.9 (*P* = 0.042), respectively).

Significant improvement in NPI-10 compared with baseline was observed from Weeks 34 to 52 in the DON5-DON10 group, and from Weeks 4 to 52 in the DON10-DON10 group, with the largest changes (mean ± SD) at Week 40 (−8.8 ± 14.9) in the DON5-DON10 group, and Week 16 (−7.3 ± 7.2) in the DON10-DON10 group. The PLA-DON10 group also showed a sustained score decrease from baseline for 52 weeks.

### Caregiver burden

**Figure 7:**
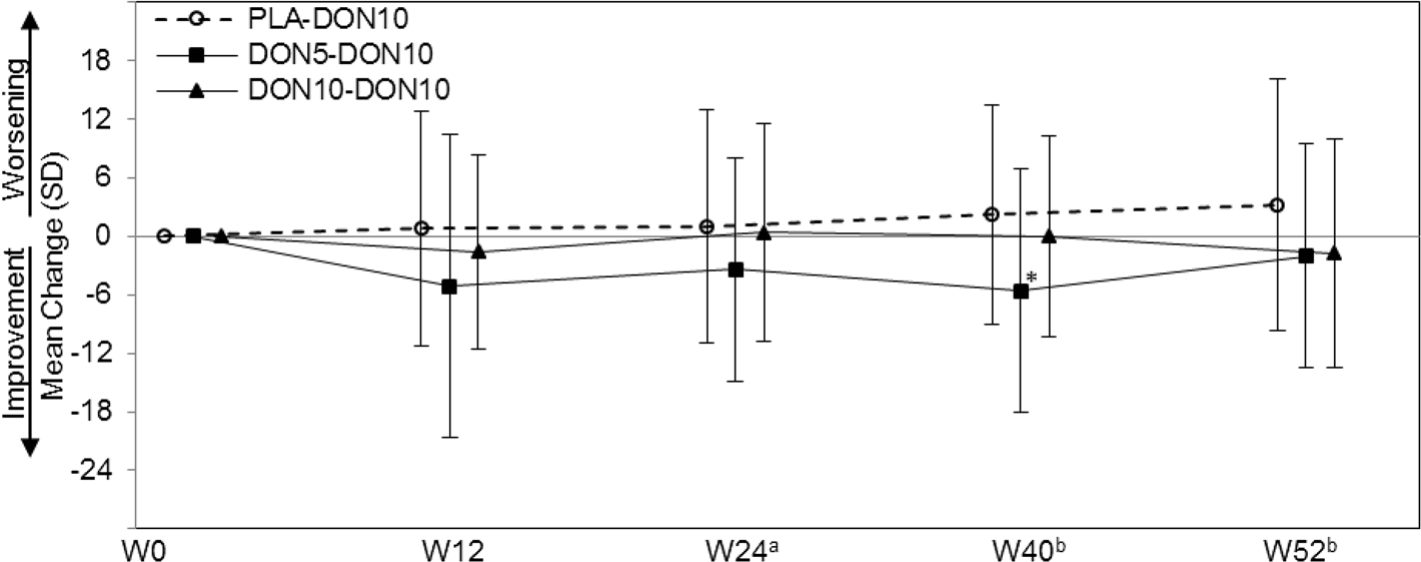
**Mean change in ZBI from baseline (FAS).** ZBI, Zarit Caregiver Burden Interview; FAS, full analysis set. ^**(a)**^PLA-DON10 group started treatment with 3 mg at Week 16, and the dose was increased to 5 mg at Week 18. ^**(b)**^PLA-DON10 and DON5-DON10 groups started treatment at 10 mg from Week 24 (dose decrease to 5 mg was allowed). *P < 0.05 (paired *t* test).

Changes in ZBI scores from baseline in each of the PLA-DON10, DON5-DON10, and DON10-DON10 groups are shown in Figure 7. The improvement was significant at Week 40 in the DON5-DON10 group, but not at any points in the PLA-DON10 and DON10-DON10 groups.

### Safety

AEs were reported by 93.8% (90 of 96) in the DON-DON10 group throughout the 52-week study period and by 89.2% (33 of 37) in the PLA-DON10 group during 36 weeks of the extension phase. Sixteen patients reported 23 serious AEs. Of these, 2 patients died because of asphyxia (PLA-DON10) or pneumonia (DON5-DON10) while receiving 10 mg, but a causal relation with the study drug was ruled out.

The incidence of AEs reported by more than 5% of the DON-DON10 group is shown in Table 2 (by 12-week intervals and total period). Major AEs with high incidence were nasopharyngitis (17.7% (17 of 96)) and parkinsonism (12.5% (12 of 96)). Treatment-related AE reported by more than 5% was only parkinsonism (10.4% (10 of 96)). All the treatment-related AEs were mild or moderate, except for 5 events (insomnia, visual hallucinations, irritability, agitation, and paranoia) reported by 2 patients in the DON5-DON10 group. The incidence of no AEs increased over time. AEs reported by the PLA-DON10 group showed a similar trend as the DON-DON10 group (Table 3).

**Table 2:** **Incidence of adverse events reported by more than 5% in the DON-DON10 group over time (SAS)**

	**DON-DON10 group (DON5-DON10** ^**a**^ **and DON10-DON10)**					
	**AE**					**Treatment-related AE** ^**b**^
**AE**	**Week 1–12**	**>Week 12–24**	**>Week 24–36**	**>Week 36**	**52 weeks**	**52 weeks**
	**(** ***n*** **= 96)**	**(** ***n*** **= 75)**	**(** ***n*** **= 72)**	**(** ***n*** **= 69)**	**(** ***n*** **= 96)**	**(** ***n*** **= 96)**
					**n (%)**	**n (%)**
Total number of incidents	65	15	7	3	90 (93.8)	46 (47.9)
Constipation	0	2	0	3	5 (5.2)	2 (2.1)
Diarrhea	2	1	1	2	6 (6.3)	1 (1.0)
Nausea	4	1	0	0	5 (5.2)	3 (3.1)
Nasopharyngitis	6	4	4	3	17 (17.7)	0
Contusion	1	2	1	3	7 (7.3)	0
Blood creatine phosphokinase increased	2	1	0	2	5 (5.2)	0
Glucose urine present	2	2	1	0	5 (5.2)	0
Decreased appetite	5	1	0	0	6 (6.3)	4 (4.2)
Muscle spasms	3	2	0	0	5 (5.2)	1 (1.0)
Parkinsonism	6	1	3	2	12 (12.5)	10 (10.4)
Insomnia	2	2	2	0	6 (6.3)	4 (4.2)

SAS, safety analysis set; AE, adverse event.

^a^Treatment with 10 mg started from Week 24.

^b^AEs for which a causal relation with the study drug was considered possible or probable.

**Table 3:** **Incidence of adverse events reported by more than 3 patients in the PLA-DON10 group over time (SAS)**

	**PLA-DON10 group** ^**a**^				
	**AE**				**Treatment-related AE** ^**b**^
**AE**	**Week 16–28**	**Week 28-40**	**Week >40**	**36 weeks**	**36 weeks**
	**(** ***n*** **=37)**	**(** ***n*** **=36)**	**(** ***n*** **=34)**	**(** ***n*** **=37)**	**(** ***n*** **=37)**
				***n*** **(%)**	***n*** **(%)**
Total number of incidents	26	7	0	33 (89.2)	22 (59.5)
Constipation	3	0	0	3 (8.1)	1 (2.7)
Diarrhea	3	0	0	3 (8.1)	2 (5.4)
Nasopharyngitis	6	3	4	13 (35.1)	0
Dizziness	3	0	0	3 (8.1)	2 (5.4)
Parkinsonism	2	1	0	3 (8.1)	3 (8.1)

SAS, safety analysis set; AE, adverse event.

^a^Treatment with 3 mg started at Week 16, and the dose was increased to 5 mg at Week 18 and to 10 mg at Week 24.

^b^AEs for which a causal relation with the study drug was considered possible or probable.

Gastrointestinal events were reported by 31.3% (30 of 96) in the DON-DON10 group. The events reported by more than 5% were diarrhea, decreased appetite (6.3% (6 of 96) each), constipation, and nausea (5.2% (5 of 96) each). All the gastrointestinal events but ileus in 1 patient (DON5-DON10, while receiving 10 mg) were mild or moderate (Table 4). In the PLA-DON10, the incidence rate was 32.4% (12 of 37). Constipation, diarrhea (8.1% (3 of 37) each), abdominal pain upper, dyspepsia, gastritis, nausea, and decreased appetite (all 5.4% (2 of 37) each) were reported by more than 5%. All these events were mild or moderate. Analyzed by 2-week intervals, the incidence rate was the highest (22.2% (8 of 36)) in the interval from Weeks 24 to 26 subsequent to the dose increase to 10 mg.

**Table 4:** **Incidence of gastrointestinal events** ^**a**^ **(SAS)**

**AE** ^**a**^	**PLA-DON10**	**DON5-DON10**	**DON10-DON10**	**DON-DON10** ^**b**^
	**(** ***n*** **=37)**	**(** ***n*** **=47)**	**(** ***n*** **=49)**	**(** ***n*** **=96)**
Subjects with any gastrointestinal events, number (%)	12 (32.4)	15 (31.9)	15 (30.6)	30 (31.3)
Abdominal discomfort	0	1 (2.1)	1 (2.0)	2 (2.1)
Abdominal pain	0	2 (4.3)	0	2 (2.1)
Abdominal pain upper	2 (5.4)	0	0	0
Constipation	3 (8.1)	1 (2.1)	4 (8.2)	5 (5.2)
Diarrhea	3 (8.1)	2 (4.3)	4 (8.2)	6 (6.3)
Dyspepsia	2 (5.4)	0	0	0
Epigastric discomfort	1 (2.7)	0	0	0
Fecal incontinence	0	1 (2.1)	0	1 (1.0)
Functional gastrointestinal disorder	0	0	1 (2.0)	1 (1.0)
Gastric ulcer	0	0	1 (2.0)	1 (1.0)
Gastritis	2 (5.4)	1 (2.1)	0	1 (1.0)
Gastrointestinal disorder	0	0	1 (2.0)	1 (1.0)
Gastroesophageal reflux disease	0	2 (4.3)	1 (2.0)	3 (3.1)
Intestinal obstruction	0	1 (2.1)	0	1 (1.0)
Nausea	2 (5.4)	3 (6.4)	2 (4.1)	5 (5.2)
Proctalgia	0	1 (2.1)	0	1 (1.0)
Vomiting	1 (2.7)	1 (2.1)	1 (2.0)	2 (2.1)
Gastroenteritis	0	1 (2.1)	0	1 (1.0)
Decreased appetite	2 (5.4)	3 (6.4)	3 (6.1)	6 (6.3)

SAS, safety analysis set; AE, adverse events. ^a^“Gastrointestinal events” included Preferred Terms (PTs) classified by the SOCs of “gastrointestinal disorders” (except for “dry mouth,” “inguinal hernia,” “dysphagia,” “toothache,” “food poisoning,” “dental caries,” “periodontal disease,” “salivary hypersecretion,” and “oral ulceration”) as well as “decreased appetite” and “gastroenteritis.

^b^DON5-DON10 and DON10-DON10 groups.

Parkinsonian symptoms were reported by 12.5% (12 of 96) in the DON-DON10 group; parkinsonism (12.5% (12 of 96)) and camptocormia (1.0% (1 of 96)) were reported (Table 5). In the PLA-DON10 group (13.5% (5 of 37)), parkinsonism (8.1% (3 of 37)), akinesia, and tremor (2.7% (1 of 37) each) were reported. None of the reported parkinsonian symptoms were severe or serious. Six events led to discontinuation or dose reduction in these patients, but all of them were recovered or relieved. UPDRS part III did not significantly increase from the baseline in any groups (Table 6). In the DON5-DON10 group, the score significantly improved throughout the study.

**Table 5:** **Incidence of parkinsonian events (SAS)**

**AE**	**PLA-DON10**	**DON5-DON10**	**DON10-DON10**	**DON-DON10** ^**a**^
	**(** ***n*** **=37)**	**(** ***n*** **=47)**	**(** ***n*** **=49)**	**(** ***n*** **=96)**
Subjects with any parkinsonian events, *n* (%)	5 (13.5)	3 (6.4)	9 (18.4)	12 (12.5)
Camptocormia	0	0	1 (2.0)	1 (1.0)
Akinesia	1 (2.7)	0	0	0
Parkinsonism	3 (8.1)	3 (6.4)	9 (18.4)	12 (12.5)
Tremor	1 (2.7)	0	0	0

SAS, safety analysis set; AE, adverse event.

^a^DON5-DON10 and DON10-DON10 groups.

**Table 6:** **Change in UPDRS part III score from baseline (SAS)**

	**PLA-DON10**				**DON5-DON10**				**DON10-DON10**			
**Evaluation points**	***n***	**Score**	**Change** ^**a**^	***P*** **value** ^**b**^	***n***	**Score**	**Change** ^**a**^	***P*** **value** ^**b**^	***n***	**Score**	**Change** ^**a**^	***P*** **value** ^**b**^
Screening	**46**	**21.4 ± 12.5**	**-**	**-**	**47**	**20.6 ± 11.9**	**-**	**-**	**49**	**19.3 ± 12.3**	**-**	**-**
Week 12	**37**	**20.1 ± 13.2**	**−1.1 ± 4.7**	***P*** **= 0.184**	*32*	*17.0 ± 11.9*	*−3.0 ± 7.6*	P = 0.032*	44	19.6 ± 13.2	0.3 ± 5.3	*P* = 0.711
Week 24	*36*	*19.1 ± 12.0*	*−1.8 ± 4.6*	P = 0.023*	*29*	*17.0 ± 11.5*	*−3.5 ± 8.3*	P = 0.029*	43	19.4 ± 14.7	−0.2 ± 8.6	*P* = 0.873
Week 40	33	19.9 ± 13.8	−2.0 ± 6.9	*P* = 0.106	25	16.1 ± 12.6	−5.0 ± 9.5	*P* = 0.014*	42	20.6 ± 15.7	0.6 ± 10.0	*P* = 0.680
Week 52	34	20.6 ± 16.0	−0.7 ± 9.4	*P* = 0.677	26	16.1 ± 13.2	−4.8 ± 10.2	*P* = 0.023*	40	21.1 ± 16.2	0.9 ± 10.0	*P* = 0.584
Week 52 (LOCF)	42	20.6 ± 14.9	−1.1 ± 8.9.	*P* = 0.431	44	19.2 ± 13.9	−1.8 ± 9.6	*P* = 0.211	48	20.1 ± 15.5	1.0 ± 9.4	*P* = 0.474

UPDRS, Unified Parkinson’s Disease Rating Scale; SAS, safety analysis set; LOCF, last observation carried forward.

Bolds indicate the period when the patients in each group took placebo.

Italics indicate the period when the PLA-DON10 and DON5-DON10 patients took 5 mg study drug (the PLA-DON10 and DON5-DON10 groups took 3 mg from Week 16 to 18 and Week 0 to 2, respectively).

The rest indicates the period when the patients took 10 mg (the DON10-DON10 group took 3 mg from Weeks 0 to 2, and 5 mg from Weeks 2 to 6).

^a^A positive value of the UPDRS part III change indicates deterioration in motor function.

^b^Student paired *t* test.

*P < 0.05.

Psychiatric events were reported by 18.8% (18 of 96) in the DON-DON10 group. Only insomnia was reported by more than 5% (6.3% (6 of 96)) (Table 7). Ten severe psychiatric events (visual hallucinations, 3; insomnia, 2; paranoia, 2; agitation, irritability, and hallucinations, 1 each) were reported by 5 patients. In the PLA-DON10 group, these events were also reported by 16.2% (6 of 37); all events were mild or moderate.

**Table 7:** **Incidence of psychiatric events** ^**a**^ **(SAS)**

**AE** ^**a**^	**PLA-DON10**	**DON5-DON10**	**DON10-DON10**	**DON-DON10** ^**b**^
	**(** ***n*** **=37)**	**(** ***n*** **=47)**	**(** ***n*** **=49)**	**(** ***n*** **=96)**
Subjects with any psychiatric events, *n* (%)	6 (16.2)	9 (19.1)	9 (18.4)	18 (18.8)
Irritability	1 (2.7)	2 (4.3)	0	2 (2.1)
Cognitive disorder	0	1 (2.1)	0	1 (1.0)
Somnolence	2 (5.4)	0	0	0
Affect lability	1 (2.7)	0	0	0
Aggression	0	0	1 (2.0)	1 (1.0)
Agitation	1 (2.7)	3 (6.4)	0	3 (3.1)
Anxiety	0	1 (2.1)	0	1 (1.0)
Apathy	0	1 (2.1)	0	1 (1.0)
Delirium	0	0	1 (2.0)	1 (1.0)
Depression	0	1 (2.1)	1 (2.0)	2 (2.1)
Disinhibition	0	1 (2.1)	1 (2.0)	2 (2.1)
Disturbance in sexual arousal	0	0	1 (2.0)	1 (1.0)
Eating disorder	0	1 (2.1)	0	1 (1.0)
Hallucination	0	1 (2.1)	2 (4.1)	3 (3.1)
Hallucination, visual	0	3 (6.4)	0	3 (3.1)
Insomnia	0	4 (8.5)	2 (4.1)	6 (6.3)
Paranoia	0	1 (2.1)	1 (2.0)	2 (2.1)
Sleep disorder	1 (2.7)	1 (2.1)	0	1 (1.0)

SAS, safety analysis set; AE, adverse event.

^a^“Psychiatric events” included Preferred Terms (PTs) classified as the SOC “Psychiatric disorders” as well as “irritability,” “cognitive disorder,” and “somnolence.”

^b^DON5-DON10 and DON10-DON10 groups.

Arrhythmic events were reported by 9.4% (9 of 96) in the DON-DON10 group, each of which was reported by less than 5% (Table 8). All the events were mild or moderate, except for loss of consciousness in 1 patient (DON10-DON10, while receiving 5 mg). In the PLA-DON10 group, 8.1% (3 of 37) of the patients reported arrhythmic events. Only loss of consciousness was reported by more than 5% (5.4% (2 of 37)). All events were mild or moderate. Four events led to discontinuation or dose reduction in these patients, but 3 of them recovered or were relieved.

**Table 8:** **Incidence of arrhythmic events (SAS)**

**AE**	**PLA-DON10**	**DON5-DON10**	**DON10-DON10**	**DON-DON10** ^**a**^
	**(** ***n*** **=37)**	**(** ***n*** **=47)**	**(** ***n*** **=49)**	**(** ***n*** **=96)**
Subjects with any arrhythmic events, *n* (%)	3 (8.1)	4 (8.5)	5 (10.2)	9 (9.4)
Atrioventricular block	0	0	2 (4.1)	2 (2.1)
Palpitations	0	1 (2.1)	0	1 (1.0)
Sinus bradycardia	0	1 (2.1)	1 (2.0)	2 (2.1)
Supraventricular extrasystoles	0	1 (2.1)	0	1 (1.0)
Ventricular extrasystoles	0	0	1 (2.0)	1 (1.0)
Electrocardiogram QT prolonged	0	1 (2.1)	0	1 (1.0)
Loss of consciousness	2 (5.4)	0	1 (2.0)	1 (1.0)
Syncope	1 (2.7)	0	0	0

SAS, safety analysis set; AE, adverse event.

^a^DON5-DON10 and DON10-DON10 groups.

Excessive decrease of systolic and diastolic blood pressure was reported by 8.4% (11 of 131) and 10.7% (14 of 131) of all the subjects, respectively. Excessive increase of blood pressure was reported by 2.3% (3 of 131) each. Abnormal change in pulse rate was reported by 3.1% (4 of 131), none of which led to any related serious AEs. Weight was decreased by 7% or more in 31.3% (41 of 131) of all the patients; only 4 of them were reported as AEs. None of the changes were reported as serious AEs.

## Discussion

The DON5-DON10 and DON10-DON10 groups showed a significant improvement on the MMSE compared with baseline for 52 weeks. The previous long-term study presented a similar treatment effect of 5 mg donepezil over 52 weeks [23]. These results suggest that improvement of cognitive impairment by donepezil at 5 mg and 10 mg is sustainable for at least 1 year in patients with DLB. In an open-label long-term study of donepezil in patients with mild to moderate AD, the improvement in MMSE was maintained until 24 weeks after administration start, and gradually waned and deteriorated afterward [32]. Considering this result in the context of a similar or faster progression in cognitive impairment in DLB than in AD [3-6], the duration during which the cognitive improvement induced by donepezil persists in patients with DLB may surpass those with AD. Although learning effects due to repeated tests possibly contributed to the improvement in the extension phase, a 1-year lasting effect of cognitive impairment is of clinical significance.

For behavioral and psychiatric symptoms, donepezil administration at any dose (5 or 10 mg) reduced the NPI-2 and NPI-10 over 52 weeks. However, similar improvement seen in the PLA-DON10 group, even from the RCT phase, makes it difficult to attribute the improvement to the study drug. It is conceivable that caregiver education about the disease and instructions on coping, which were likely given at the beginning of and during the study, affected the behavioral and psychiatric symptoms. However, because it is unlikely to last long, such an effect on the symptom improvement may be replaced or enhanced by donepezil after treatment initiation and may lead to a 1-year lasting improvement, even in the PLA-DON10 group.

With regard to the effect of dose increment in the DON5-DON10 group, although no significant improvement due to the dose increment was detected either in MMSE score or in NPI-2 score as a whole, the subgroup either with an MMSE change of <3 points or with a NPI-2 change of <30% from the baseline at Week 24 showed an improvement after the dose increment. There may be a range of doses at which the maximum improvement can be obtained, and 5 mg can provide a sufficient effect to some patients. The expected further improvement by increasing to 10 mg may allow recommendation for a dose increase to 10 mg based on the individual safety when 5 mg is insufficient.

After Week 24, 18 patients experienced a dose reduction from 10 mg to 5 mg. Because MMSE scores remained above the baseline at all times, without deterioration of more than 0.7 points, the effects can be maintained even with a reduction to 5 mg. When intolerable at 10 mg, treatment could effectively be continued by dose reduction to 5 mg.

No great difference was observed in the occurrence of AEs due to the length of the administration period. Thus, the possibility of delayed onset of AEs with long-term treatment seems low. Most of the treatment-related AEs were mild or moderate, and only parkinsonism had an incidence of 5% or more. Of the 107 patients who continued the treatment beyond Week 24, dosage was reduced in 21 (19.6%) of patients. The main adverse events leading to dose reduction were gastrointestinal, psychiatric, and parkinsonian symptoms. All of these resolved or were relieved after dose reduction, and did not lead to discontinuation after the reduction. Gastrointestinal events are well-known adverse events of ChEIs. Gastrointestinal events most frequently reported by the patients who received 10 mg of donepezil in the 52-week study in AD patients were diarrhea (12.7%), nausea (12.2%), and vomiting (10.1%) [33]; the equivalent incidences of these in the present study in patients with DLB were lower. A slight increase in the incidence after a dose increase from 5 to 10 mg suggests the need to pay attention to the occurrence of gastrointestinal events on dose increase. However, this comparison, the present result of mostly mild to moderate severity and the absence of an increasing trend in the incidence over time support a low risk for clinically significant gastrointestinal symptoms.

Another AE of specific concern is parkinsonism; donepezil may induce or exacerbate extrapyramidal symptoms, which are threatening for DLB patients in whom parkinsonism occurs frequently. However, none of the reported parkinsonian symptoms was severe or serious. Neither the incidence nor UPDRS part III scores were inclined to increase over time, representing no notable deterioration over time. Psychiatric events were not considered to be notable safety concerns, according to their incidence (including lower rate in 10 mg group in the RCT). Arrhythmic events require particular attention, based on the incidence of 9.0% (12 of 133) of all the included patients and 3 cases of loss of consciousness, one of which was severe. In the RCT phase, the incidence of arrhythmic events did not clearly tend to increase in the active groups (placebo, 5, and 10 mg: 4.3%, 4.3%, and 6.1%, respectively). In the extension phase, the incidence by 12-week intervals did not exceed the incidence in the placebo group during the RCT phase. As loss of consciousness reported by 1 patient in the placebo group during the RCT phase is certainly attributed to the disease itself, those reported in patients who received donepezil might not be necessarily attributed to donepezil.

Another safety event to be noted is abnormal weight loss, which was reported in a substantial proportion of patients. However, it was mostly self-limited and not serious, as it was rarely recognized to be an adverse event.

The findings suggest that no major concerns exist regarding the safety or tolerability profile of long-term administration of donepezil at up to 10 mg. Safe and tolerable treatment can be assured by alerting the patients and their caregivers about the occurrence of parkinsonism and gastrointestinal or arrhythmic symptoms and managing the risks for such events by reducing the dose.

The major limitations include the short duration (12 week) of the RCT phase and the open-label design of the extension phase as well as the small sample size. Because of the progressive nature of this disease and the increasing caregiver stress, it would be difficult to enroll patients with DLB in a long-term placebo-controlled trial. For these reasons, the long-term efficacy and safety of 10 mg of donepezil over 5 mg or placebo cannot be stated assertively.

## Conclusions

The open-label long-term administration of donepezil at 10 mg/day improved impaired cognitive function for up to 52 weeks in patients with DLB without increasing the risk of clinically significant safety events.

## Additional file

Additional file 1:
**List of all institutional review board.**

## Abbreviations

AD: Alzheimer disease
AE: adverse event
ANCOVA: analysis of covariance
ChAT: choline acetyltransferase
ChEI: cholinesterase inhibitor
DLB: dementia with Lewy bodies
FAS: full analysis set
LOCF: last observation carried forward
MMRM: mixed-effect model for repeated measures
MMSE: Mini-Mental State Examination
NPI: neuropsychiatric Inventory
PPS: per protocol set
RCT: randomized, double-blind, placebo-controlled
SAS: safety analysis set
SD: standard deviation
UPDRS: Unified Parkinson’s Disease Rating Scale
ZBI: Zarit Caregiver Burden Interview
