# Supplementary material for: Long-term donepezil use for dementia with Lewy bodies: results from an open-label extension of Phase III trial
Source: Alzheimers Res Ther. 2015 Feb 3;7(1):5. doi: 10.1186/s13195-014-0081-2 (PMC4338564; doi:10.1186/s13195-014-0081-2)
Supplement: Additional file 1: — List of all institutional review board. [file 13195_2014_81_MOESM1_ESM.pdf]

Institutional review boards (IRBs):

Himeji Central Hospital IRB (Himeji Central Hospital), Omuta Hospital IRB (Omuta Hospital), Shinozuka Hospital IRB (Shinozuka Hospital), Shinagawa Clinic IRB (Uematsu Neurological Clinic), Toyokawa City Hospital IRB (Toyokawa City Hospital), Hyogo Brain and Heart Center IRB (Hyogo Brain and Heart Center), Yoka Hospital IRB (Yoka Hospital), Himorogi Psychiatric Institute IRB (Miyoshi Neurology Clinic, Matsubara Hospital, Shirai Hospital, Kudoh Chiaki Hospital, Nishi-Kobe Medical Center, and Okumura Clinic), Juntendo Tokyo Koto Geriatric Medical Center IRB (Juntendo Tokyo Koto Geriatric Medical Center), Gunma University Hospital IRB (Gunma University Hospital), Saitama Medical University IRB (Saitama Neuropsychiatric Institute), Shinagawa East One Medical Clinic IRB (Takesato Hospital and Kurumi Clinic), Tokyo Medical University Hospital IRB (Tokyo Medical University Hospital), Osaka Red Cross Hospital IRB (Osaka Red Cross Hospital), Hiroshima-Nishi Medical Center IRB (Hiroshima-Nishi Medical Center), Fukuoka University Hospital IRB (Fukuoka University Hospital), Kikuti National Hospital IRB (Kikuti National Hospital), Osaka University Hospital IRB (Osaka University Hospital), Nagasaki Kita Hospital IRB (Nagasaki Kita Hospital), Sukoyaka-silver Hospital IRB (Sukoyaka-silver Hospital), Kanto Central Hospital IRB (Kanto Central Hospital), Takeda General Hospital joint IRB (Takeda General Hospital), Susaki Kuroshio Hospital, Tano Hospital, and Takeshita Hospital joint IRB (Nanohana Clinic), University of Fukui Hospital IRB (University of Fukui Hospital), Obitsu Sankei Hospital IRB (Kawashima Neurology Clinic), Ina Central Hospital IRB (Ina Central Hospital), Tenryu Hospital IRB (Tenryu Hospital), MEDOC Medical Dock & Clinic IRB (MEDOC Medical Dock & Clinic), Nagoya Ekisaikai Hospital IRB (Nagoya Ekisaikai Hospital), Asakayama General Hospital IRB (Asakayama General Hospital), Kinki University Hospital IRB (Sakai Hospital Kinki University Faculty of Medicine), Tsubame Rosai Hospital IRB (Tsubame Rosai Hospital), Yuge Hospital IRB (Yuge Hospital), Keihin central clinic, Hisamitsu clinic and Masabayashi clinic joint IRB (Mishima Hospital and Kawase Neurology Clinic), Chubu Rosai Hospital IRB (Chubu Rosai Hospital), Utano Hospital IRB (Utano Hospital), Heiwadai Hospital IRB (Keimei Memorial Hospital), Ageo Central General Hospital IRB (Ageo Central General Hospital), Ebara Hospital IRB (Ebara Hospital), Shizuoka Institute of Epilepsy and Neurological Disorders IRB (Shizuoka Institute of Epilepsy and Neurological Disorders), Sapporo Ryokuai Hospital IRB (Oe Hospital), The Japanese Association for the Promotion of State-of-the-Art in Medicine IRB (Hospital Bando), Chiba-East Hospital IRB (Chiba-East Hospital), Takamatsu Hospital IRB (Takamatsu Hospital), Osaka City Kosaiin Hospital IRB (Osaka City Kosaiin Hospital), NPO Osaka Clinical Trials Network IRB (Izumino Hospital), Iwate Medical University Hospital IRB (Iwate Medical University Hospital), Kure Medical Center IRB (Kure Medical Center), Kobe City Medical Center West Hospital IRB (Kobe City Medical Center West Hospital), Kumamoto University Hospital IRB (Kumamoto University Hospital), and Oita University Hospital IRB (Oita University Hospital).
